# Risk factor analysis and optimal cutoff value selection of PSAD for diagnosing clinically significant prostate cancer in patients with negative mpMRI: results from a high-volume center in Southeast China

**DOI:** 10.1186/s12957-024-03420-7

**Published:** 2024-05-28

**Authors:** Shen Lin, Wubin Jiang, Jiafeng Ding, Sida Hao, Hong Chen, Liping Xie, Xiangyi Zheng

**Affiliations:** 1 Department of Urology, the First Affiliated Hospital, Zhejiang University School of Medicine, Hangzhou, Zhejiang 310003 China; 2 Department of Urology, Taizhou Hospital of Zhejiang Province affiliated to Wenzhou Medical University, Taizhou, Zhejiang China; 3 Department of Urology, Lishui Hospital of Zhejiang University, No. 289 Kuocang Road, Lishui, Zhejiang 323000 China

**Keywords:** Prostate cancer, PSAD, Negative mpMRI, Prostate biopsy, Risk factors

## Abstract

**Background:**

Multi-parametric magnetic resonance imaging (mpMRI) is a diagnostic tool used for screening, localizing, and staging prostate cancer. Patients with Prostate Imaging Reporting and Data System (PI-RADS) score of 1 and 2 are considered negative mpMRI, with a lower likelihood of detecting clinically significant prostate cancer (csPCa). However, relying solely on mpMRI is insufficient to completely exclude csPCa, necessitating further stratification of csPCa patients using biomarkers.

**Methods:**

A retrospective study was conducted on mpMRI-negative patients who underwent prostate biopsy at the First Affiliated Hospital of Zhejiang University from January 2022 to June 2023. A total of 607 patients were included based on inclusion and exclusion criteria. Univariate and multivariate logistic regression analysis were performed to identify risk factors for diagnosing csPCa in patients with negative mpMRI. Receiver Operating Characteristic (ROC) curves were plotted to compare the discriminatory ability of different Prostate-Specific Antigen Density (PSAD) cutoff values for csPCa.

**Results:**

Among the 607 patients with negative mpMRI, 73 patients were diagnosed with csPCa. In univariate logistic regression analysis, age, PSA, f/tPSA, prostate volume, and PSAD were all associated with diagnosing csPCa in patients with negative mpMRI (*P* < 0.05), with PSAD being the most accurate predictor. In multivariate logistic regression analysis, f/tPSA, age, and PSAD were independent predictors of csPCa (*P* < 0.05). PSAD cutoff value of 0.20 ng/ml/ml has better discriminatory ability for predicting csPCa and is a significant risk factor for csPCa in multivariate analysis.

**Conclusion:**

Age, f/tPSA, and PSAD are independent predictors of diagnosing csPCa in patients with negative mpMRI. It is suggested that patients with negative mpMRI and PSAD less than 0.20 ng/ml/ml could avoid prostate biopsy, as a PSAD cutoff value of 0.20 ng/ml/ml has better diagnostic performance than the traditional cutoff value of 0.15 ng/ml/ml.

**Supplementary Information:**

The online version contains supplementary material available at 10.1186/s12957-024-03420-7.

## Introduction

Prostate cancer (PCa) is the most common male genitourinary malignancy. In GLOBOCAN Statistics 2020, there were 1,414,529 new cases of prostate cancer worldwide, resulting in 375,304 deaths [1]. The 2023 U.S. Cancer Statistics revealed that prostate cancer, lung cancer, and colorectal cancer were the most prevalent cancers among males, with prostate cancer accounting for the highest proportion at 29% [2]. In February 2024, China’s National Cancer Center updated its statistics, revealing a trend of cancer incidence approaching that of Western countries. In 2022, there were 134,200 new cases of prostate cancer nationwide, with an age-standardized incidence rate of 9.68 per 100,000 people. 47,500 people died from prostate cancer, with an age-standardized mortality rate of 3.26 per 100,000 people [3]. This increase can be attributed to the widespread adoption of prostate-specific antigen (PSA) screening and the application of multi-parameter magnetic resonance imaging (mpMRI) in recent years.

Prostate mpMRI is a useful imaging technique for the screening, locating, and staging of prostate cancer. It includes multiple sequences such as T1-weighted imaging (T1WI), T2-weighted imaging (T2WI), diffusion-weighted imaging (DWI), dynamic contrast-enhanced imaging (DCEI) [4]. The introduction of the Prostate Imaging Reporting and Data System (PI-RADS) has enhanced the diagnostic accuracy of prostate cancer. Subsequent versions, PI-RADs v2 and v2.1, have aimed to mitigate specific subjective scoring criteria, thereby further improving the standardization of reporting [5, 6]. Recent clinical studies have demonstrated the precision of PI-RADS in identifying clinically significant prostate cancer (csPCa) and providing guidance for the clinical practice of prostate biopsy [7].

Patients with PI-RADS scores 1 and 2, indicating negative mpMRI, can avoid prostate biopsy due to a reduced likelihood of detecting csPCa. However, Sathianathen et al. stated that there is still a 5-15% chance of missing csPCa in patients with negative mpMRI [7]. Further risk stratification tools are needed in conjunction with mpMRI to determine whether a prostate biopsy should be performed.

Prostate-specific antigen density (PSAD) can be combined with PI-RADS to assess patients, achieving better stratification of individuals enrolled in Active Surveillance [8] and more accurate prediction of csPCa [9–11]. The EAU (European Association of Urology) recommends a PSAD cutoff of 0.15 ng/ml/ml, indicating that patients above this value should have a prostate biopsy. However, the effectiveness and accuracy of this cutoff have not been established. Pellegrino et al. pointed out that this cutoff value originated from clinical studies conducted in the previous century when patients had not undergone mpMRI before biopsy [12]. The current clinical studies on PI-RADS and PSAD show significant variations in recommended PSAD thresholds due to differences in study design, cancer screening criteria, and mpMRI protocols [10, 11, 13–16].

This study retrospectively analyzed patients who underwent prostate biopsy at the First Affiliated Hospital of Zhejiang University School of Medicine (FAHZU) between January 1, 2022, and June 30, 2023, and had negative mpMRI results. A logistic regression model was utilized to analyze independent predictors of csPCa among patients with negative mpMRI results. The diagnostic efficacy of different PSAD cutoff values was computed to select an appropriate cutoff value that could potentially avoid biopsy. This study provides support for urologists in making clinical decisions regarding prostate biopsy for patients with negative MRI results based on the Chinese population.

## Methods

### Patients

Clinical data of 4223 patients suspected of prostate cancer undergoing prostate biopsy from January 2022 to June 2023 were collected through the medical system of the First Affiliated Hospital, Zhejiang University School of Medicine. Based on the inclusion and exclusion criteria, we included 607 biopsy-naïve patients with negative mpMRI. This research has been approved by the Ethics Committee of the First Affiliated Hospital, Zhejiang University School of Medicine.

Inclusion Criteria:

1. Patients who receiving prostate biopsy at our hospital, with complete clinical data.
2. mpMRI was performed within four weeks before the biopsy.
3. Patients with PI-RADS v2.1 score ≤ 2.

Exclusion criteria:

1. Patients with missing clinical data.
2. Patients who did not receive mpMRI within 4 weeks prior to the biopsy.
3. Patients with PI-RADS v2.1 score > 2.
4. Patients with a history of prostate biopsy or already diagnosed with prostate cancer.
5. Patients with a history of prostate surgical treatment.

### Data collection

The collected data includes age, height, weight, prostate volume, PSA, free PSA(fPSA), f/tPSA, PI-RADS score, biopsy results, digital rectal examination, and the comorbidities of the patients. PSA-related data were collected within the four weeks preceding the biopsy.

All included patients underwent mpMRI (Signa HDX 3.0 T, GE Healthcare, US) performed at 3.0-T with an eight-channel phased-array body coil. Two experienced radiologists independently reviewed the images without knowledge of the patient’s medical history and pathological results. The PI-RADS v2.1 scoring system was used to report the mpMRI results [5]. Prostate volume was calculated on mpMRI using the standard ellipsoid formula.

A Transrectal ultrasound (TRUS)-guided transperineal 12-core biopsy was performed for each patient. Using an 18-gauge biopsy needle, ten to twelve core biopsies was taken from the peripheral gland, bilateral from apex to base, as far posterior and lateral as possible. Additional cores was obtained from DRE/TRUS suspicious areas [17].

All systematic biopsies were performed using an Esaote MyLabTMClassC ultrasound unit and an 8 MHz transrectal biplane TRT33 probe (Esaote, Italy).

Pathological assessment was conducted by a group of experienced pathologists following the 2014 International Society of Urological Pathology (ISUP) guidelines [18].

### Definition of negative mpMRI and csPCa

Patients with a PI-RADS score of 1 or 2 were considered negative mpMRI.

The standard for csPCa has not been unified yet. The criteria used in the literature include: ISUP ≥ 2; ISUP 1 with maximum cancer core length ≥ 6 mm and ISUP grade group ≥ 2 and ISUP ≥ 3 [19–23]. PI-RADS v2.1 guidelines suggest that csPCa should possess at least one of the following characteristics: (1) Gleason score ≥ 3 + 4; (2) Tumor volume > 0.5 cc; (3) mpMRI indicating tumor with extraprostatic extension [5]. Sathianathen et al.‘s systematic review on the predictive value of mpMRI included 42 studies, and the majority of studies defined csPCa as ISUP ≥ 2.

Referring to the above studies, csPCa in this study is defined as ISUP ≥ 2.

### Statistical analyses

Frequency and percentage were used to report the categorical data. Pearson’s chi-square test was employed to assess differences between categorical variables. The mean and standard deviation, or the median and interquartile range, describe continuous variables. The normality of continuous variables was evaluated using the Kolmogorov-Smirnov test. Students’ t-test or Mann-Whitney U test were used to compare continuous variables. Logistic regression is used to assess whether variables are independent predictors. A p-value less than 0.05 was considered statistically significant. Statistical analysis was performed using R 4.2.3 software.

## Results

**Fig. 1:**
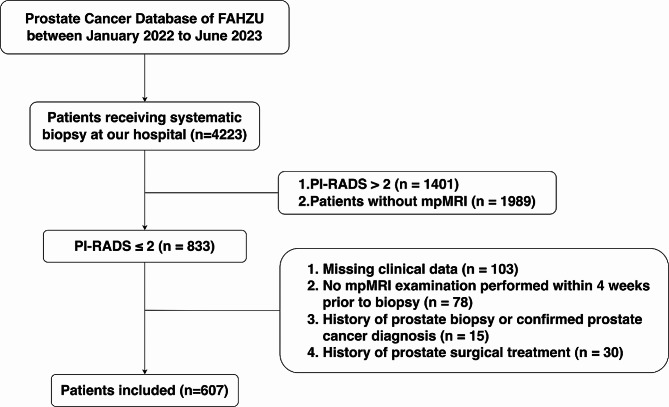
Patients selection flow chart

The study ultimately included 607 patients who underwent prostate biopsy with negative mpMRI results (Fig. 1; Table 1). Of these, 73 cases (12.03%) were classified as csPCa (ISUP ≥ 2), 97 (15.98%) as non-clinically significant prostate cancer (non-csPCa, ISUP = 1), and 437 (71.99%) as benign prostatic hyperplasia. Significant statistical differences (*P* < 0.05) were observed between the csPCa and non-csPCa groups in age, prostate volume (PV), PSA, and PSAD, suggesting that these characteristics may serve as potential predictive factors for identifying csPCa.

Due to the limited number of positive findings and presence of missing values in digital rectal examination (DRE), no significant differences were observed between the two groups. DRE has limited predictive accuracy for biopsy results, and it shows no substantial correlation with ISUP Grading Groups (ISUP GG), thus excluding it from further analysis (Supplementary Fig. 1).

**Table 1:** Baseline Characteristics of Patients with Negative mpMRI.

	Non-CsPCa(*N* = 534)	CsPCa(*N* = 73)	*P* value
Age(year)	65.0 [60.0;70.0]	70.0 [66.0;76.0]	<0.001
Height(cm)	170 [165;173]	170 [165;172]	0.370
Weight(Kg)	68.0 [62.0;75.0]	67.0 [60.0;73.0]	0.487
PI-RADS (V2.1):			0.402
1	3 (0.56%)	1 (1.37%)	
2	531 (99.4%)	72 (98.6%)	
Prostate Volume (ml)	51.5 [38.6;69.2]	37.6 [29.4;49.4]	<0.001
Biopsy results:			
Benign prostate	437 (81.84%)	0 (0.00%)	
ISUP Grading Groups:			
1	97 (18.16%)	0 (0.00%)	
2	0 (0.00%)	33 (45.2%)	
3	0 (0.00%)	33 (45.2%)	
4	0 (0.00%)	4 (5.48%)	
5	0 (0.00%)	3 (4.11%)	
PSA (ng/mL)	8.26 [5.98;11.2]	11.2 [7.97;16.4]	<0.001
fPSA(ng/mL)	1.53 [0.95;2.24]	1.46 [1.17;2.20]	0.485
f/tPSA	0.18 [0.13;0.25]	0.13 [0.10;0.20]	<0.001
Hypertension (HPT):			0.161
No HPT	333 (62.7%)	39 (53.4%)	
HPT	198 (37.3%)	34 (46.6%)	
Diabetes Mellitus (DM):			0.117
No DM	491 (92.5%)	63 (86.3%)	
DM	40 (7.53%)	10 (13.7%)	
Cardiovascular Disease (CVD)			1.000
No CVD	506 (95.3%)	70 (95.9%)	
CVD	25 (4.71%)	3 (4.11%)	
PSAD(ng/ml/ml)	0.16 [0.11;0.23]	0.31 [0.19;0.44]	<0.001
Digital Rectal Examination:			0.073
Negative	157 (98.7%)	20 (90.0%)	
Positive	2 (1.26%)	2 (9.09%)	
BMI	23.7 [22.0;25.4]	24.0 [21.4;25.6]	0.820

**Fig. 2:**
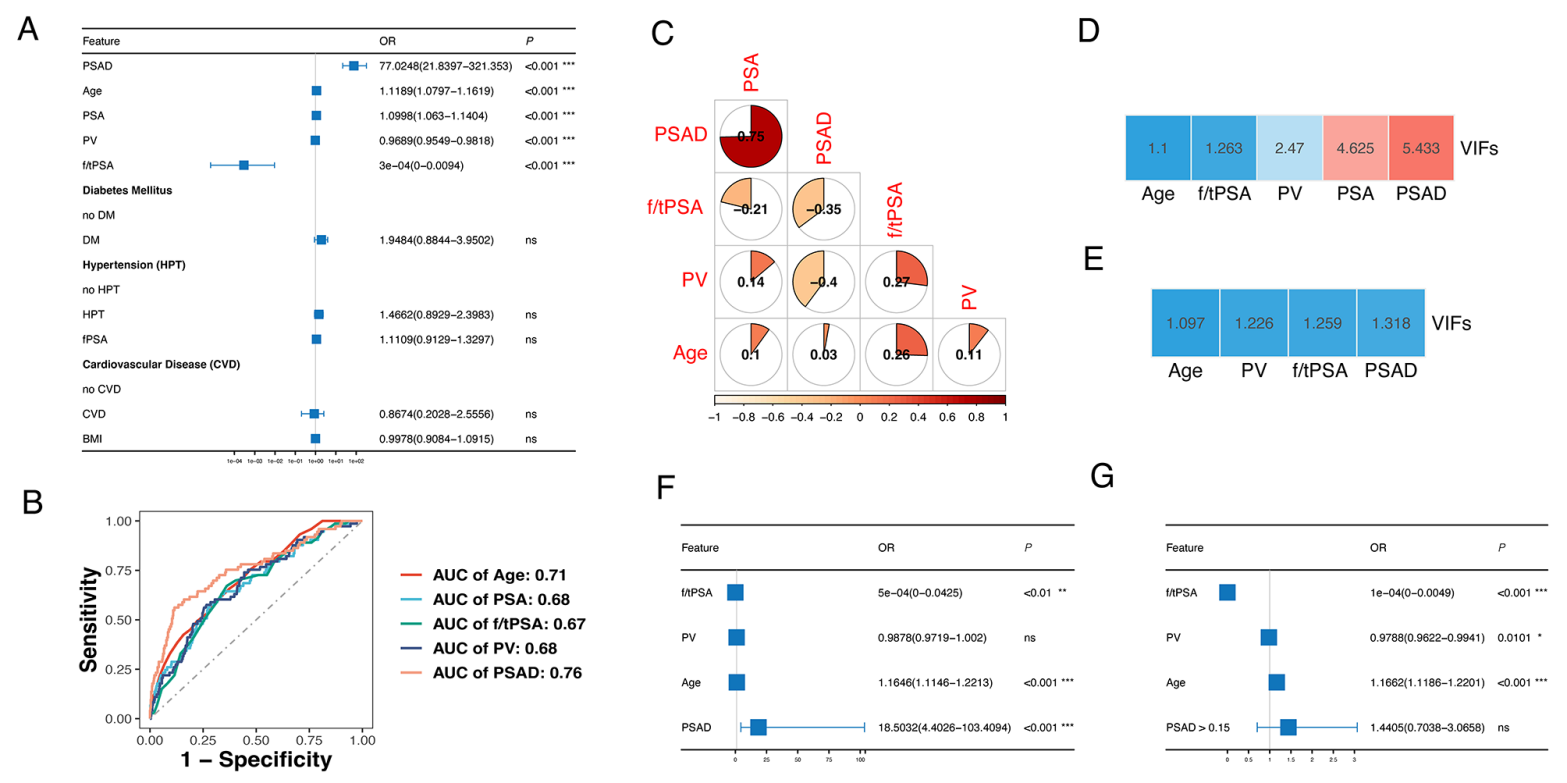
Univariate and multivariate logistics regression analysis for identifying csPCa in mpMRI-negative patients. **A**: Univariate logistic regression for potential predictors of csPCa in mpMRI -negative patients. **B**: ROC curves for predicting csPCa using clinical features. **C**: The pie chart illustrating the pairwise correlations among variables. D-E: VIF (Variance Inflation Factor) analysis including PSA (**D**) and excluding PSA (**E**) for clinical features. **F**: Forest plot displaying the OR values of variables in multivariate logistic regression. **G**: Forest plot displaying the OR values of variables in multivariate logistic regression including PSAD>0.15 ng/ml/ml

Univariate logistic regression analysis indicated that older age, higher PSA levels, lower f/tPSA ratio, smaller prostate volume, and higher PSAD were all significantly associated with csPCa on biopsy (Fig. 2A). These findings suggest that these characteristics may serve as potential predictive factors for csPCa among patients with negative mpMRI results. Subsequently, we plotted ROC curves for each factor and calculate the area under the curve (AUC). PSAD was identified as the most accurate predictor (Fig. 2B).

Before conducting the multivariate regression analysis, we observed that PSAD is derived by dividing PSA by PV, potentially introducing collinearity into the model. Therefore, we computed the correlation between each pair of variables (Fig. 2C). The results indicated a correlation coefficient greater than 0.7 between PSAD and PSA, suggesting the presence of collinearity affecting the model. Using linear regression analysis to calculate the variance inflation factor (VIF), we observed high VIF values for PSAD and PSA, with PSAD’s VIF exceeding 5 (Fig. 2D). Since PSAD exhibited the highest AUC in the ROC curve analysis, we hypothesize that the elevated VIF of PSAD is due to its strong correlation with PSA, leading to higher collinearity with other variables. We have decided to exclude PSA and recalculate the VIF. As a result, we observed a decrease in the VIF values for all variables, within a reasonable range (Fig. 2E).

Finally, age, f/tPSA, PV, and PSAD were included in the multivariate logistic regression analysis. The results indicated that age, f/tPSA and PSAD were independent predictors for csPCa (Fig. 2F). Considering the EAU guidelines for prostate cancer, which suggest that in mpMRI-negative patients, urologists could jointly decide with patients to avoid prostate biopsy if PSAD is less than 0.15 ng/ml/ml. To comply with the application of PSAD, we transformed PSAD into a binary variable using a cutoff value of 0.15 ng/ml/ml and incorporated it into the multivariate analysis. The results indicate that PSAD exceeding 0.15 ng/ml/ml l is not an significant risk factor for csPCa (Fig. 2G). Therefore, it may be necessary to select a PSAD cutoff value more suitable for the Chinese patient population.

.

**Fig. 3:**
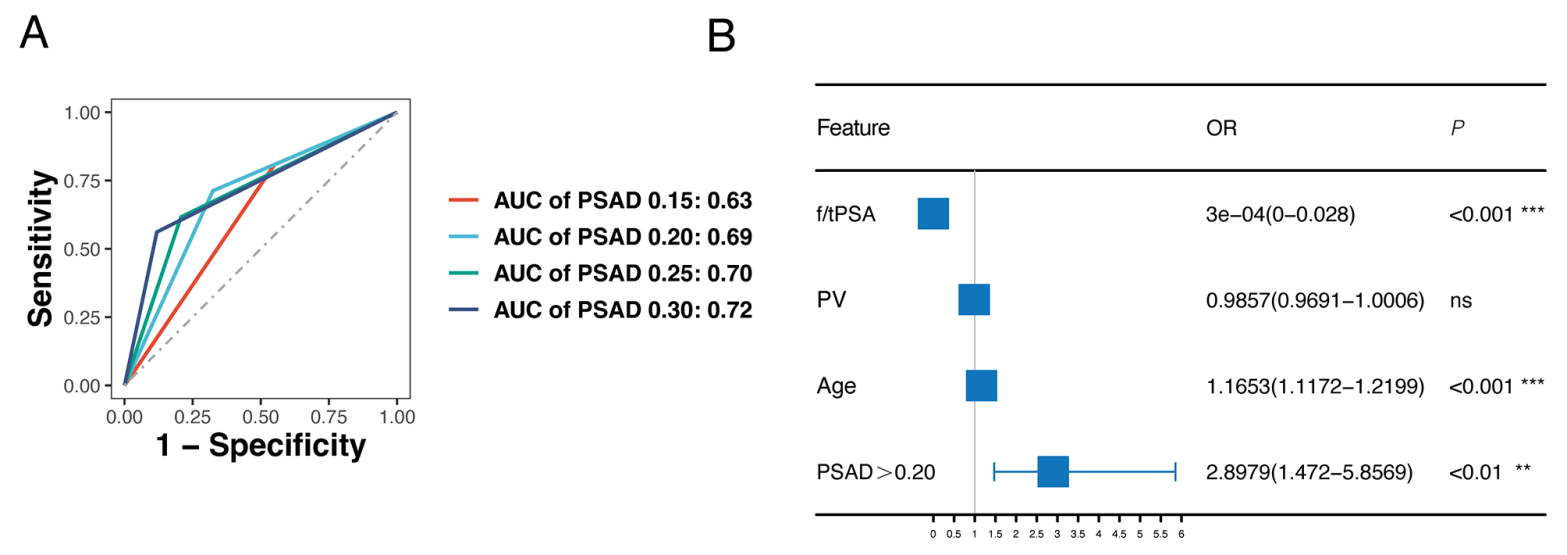
The ROC curve and multivariate logistics analysis for PSAD: 0.20 ng/ml/ml. (**A**) ROC curves for predicting csPCa using PSAD: 0.20 ng/ml/ml. (**B**) Forest plot displaying the OR values of PSAD: 0.20 ng/ml/ml in multivariate logistic regression

To compare the performance of different PSAD cutoff values, PSAD was transformed into binary variables using cutoff values of 0.15, 0.20, 0.25, and 0.30 ng/ml/ml. ROC curves were plotted for different PSAD cutoff values, and the corresponding AUC values were calculated (Fig. 3A). Negative predictive values and positive predictive values were calculated and are presented in Table 2. The results indicate that when the cutoff value of PSAD is 0.15 ng/ml/ml, PSAD does not effectively discriminate csPCa. However, when the cutoff value of PSAD increases to 0.20 ng/ml/ml, it can accurately identify csPCa while minimizing the risk of missed diagnoses. We incorporated PSAD: 0.20 ng/ml/ml, age, f/tPSA, and PV into the multivariate analysis, and observed that PSAD more than 0.20 ng/ml/ml is a crucial risk factor for csPCa (Fig. 3B). This suggests that the cutoff value of 0.20 ng/ml/ml has superior predictive value compared to the PSAD: 0.15 ng/ml/ml.

Therefore, we propose that patients with negative mpRMI and PSAD less than 0.20 ng/ml/ml may avoid prostate biopsy.

**Table 2:** The negative predictive value, and positive predictive value for PSAD cutoff values

PSAD (ng/ml/ml)	Sensitivity	Speciality	AUC	NPV	PPV	False Negative (*n*)	True Negative (*n*)
0.15	0.808	0.450	0.629	0.945	0.168	14	239 (38)*
0.20	0.712	0.676	0.694	0.945	0.232	21	359 (61) *
0.25	0.616	0.793	0.705	0.938	0.290	28	421 (71) *
0.30	0.562	0.881	0.721	0.936	0.394	32	468 (81) *

*The number in brackets represents the count of patients with ISUP GG 1.

## Discussion

With ongoing advancements in prostate cancer diagnosis, targeted biopsies guided by mpMRI or Prostate Specific Membrane Antigen (PSMA) PET/CT have steadily increased the detection rate of prostate cacner, reducing unnecessary biopsies [24, 25].

The predictive value of PI-RADS in the early diagnosis of PCa has been confirmed by numerous studies. For patients with PI-RADS scores of 1–2, the risk of csPCa is relatively low. Hansen et al. recruited 236 patients with PI-RADS scores of 1–2, among whom 47 cases of csPCa were detected after transperineal biopsy [26]. A systematic review by Sathianathen et al. found that depending on the criteria used for mpMRI negativity and csPCa definition, the negative predictive value of negative mpMRI ranged from 86.8 to 97.1% [7]. These findings suggest that a negative mpMRI does not completely rule out csPCa, and missing any case of csPCa can lead to serious consequences. Hence, identifying risk factors associated with diagnosing csPCa in mpMRI-negative patients holds crucial clinical significance.

Based on a retrospective analysis of 607 mpMRI-negative patients from the First Affiliated Hospital of Zhejiang University, this study identified independent predictive factors for diagnosing csPCa as f/tPSA, prostate volume, age, and PSAD, among which PSAD emerged as the strongest predictor. PSAD has been widely utilized as a biomarker for prostate cancer identification. Teoh et al.‘s study found that a PSAD value of 0.12 ng/ml/ml in the Chinese population yielded a sensitivity of 94.5%, NPV of 92.7%, and an AUC of 0.823 in diagnosing PCa, with PSAD being a significant risk factor in multivariate regression analysis (OR: 6.22, 95% CI: 4.20–9.22) [27]. Other studies focusing on the Chinese population have also demonstrated similar findings [28, 29], indicating PSAD as a robust tumor marker for prostate cancer.

In patients with negative mpMRI findings, the EAU Prostate Cancer Guidelines recommend a PSAD cutoff of 0.15 ng/ml/ml to avoid prostate biopsy. Luiting et al.‘s study included 467 patients from the PRIAS (The Prostate cancer Research International: Active Surveillance) study with negative mpMRI [13]. Buisset et al. enrolled 503 mpMRI-negative patients from a single center in France [14], while Ma et al.‘s study included 150 mpMRI-negative patients from the United States [16]. These studies all identify PSAD > 0.15 ng/ml/ml as a risk factor for csPCa. Norris et al. suggested that using a lower PSAD cutoff of 0.10 ng/ml/ml can reduce the false-negative rate for diagnosing csPCa to 3% [30].

In studies primarily involving Chinese patients, E et al.‘s research included 335 mpMRI-negative patients, recommending prostate biopsy for patients with PSAD more than 0.18 ng/ml/ml [31]. Zhang et al.‘s study, which incorporated 240 MRI-negative patients from West China Hospital, suggests that PSAD < 0.20 ng/ml/ml significantly increases the NPV for diagnosing csPCa [11]. However, these studies had relatively small sample sizes and did not incorporate PSAD as a dichotomous variable into multivariable regression to further test its statistical significance. Therefore, based on a cohort of 607 mpMRI-negative patients from our institution, this study found that PSAD more than 0.15 ng/ml/ml is not an independent risk factor for diagnosing csPCa.

It appears that PSAD values for Chinese patients may be slightly higher compared to other populations. We examined several potential cutoff values greater than 0.15 ng/ml/ml for PSAD. The results suggest that a PSAD value of 0.20 ng/ml/ml has an AUC value of around 0.7 and the highest negative predictive value. This cutoff ensures minimal underdiagnosis while accurately identifying csPCa. Consistent with previous research findings, it can be observed that the recommended threshold for avoiding prostate biopsy in Chinese mpMRI-negative patients tends to be higher. This may be related to differences in prostate cancer incidence rates, which are significantly lower in China compared to Western developed countries. Lower PSAD cutoff values are needed to avoid underdiagnosis in western patients [32]. Additionally, the PV of Chinese patients is generally smaller than that of Western Caucasian patients [27]. In Buisset et al.‘s study, the median prostate volume for mpMRI-negative patients was 59.89 ml (interquartile range: 40 to 70 ml). In this study, non-csPCa patients had a median prostate volume of 51.5 ml (interquartile range: 38.6 to 69.2 ml), while csPCa patients had a median prostate volume of 37.6 ml (interquartile range: 29.4 to 49.4 ml), indicating larger prostate volumes in Caucasian patients [14]. The smaller prostate volume in Chinese patients results in higher PSAD cutoff values compared to the Caucasian population. However, further prospective multicenter studies are needed to confirm this observation.

Another possible reason is the varying accuracy of mpMRI. Pellegrino et al. computed the negative likelihood ratio based on reported sensitivities and specificities of mpMRI from different literatures [12]. However, the differences in inclusion and exclusion criteria for patients, variation in biopsy histories, and diverse mpMRI scoring systems across studies have resulted in a wide range of negative likelihood ratios for mpMRI (0.08–0.34). Through statistical analysis, it was determined that when the negative likelihood ratio of mpMRI is relatively poor, approaching 1, a PSAD value of 0.15 ng/ml/ml may be a reasonable threshold for intervention recommendation. Conversely, when the negative likelihood ratio of mpMRI is better, approaching 0, higher PSAD thresholds are needed to avoid unnecessary biopsies.

This study has certain limitations. Firstly, the identification of csPCa relies on the pathological results from systematic biopsy rather than post-prostatectomy specimen pathology. The most widely used biopsy approach is the 12-core systematic biopsy, which may miss some tumors, particularly in larger prostates. Different biopsy methods can impact tumor detection rates, and anterior prostate biopsy has a higher ability to detect anterior tumors [33]. Secondly, there is ongoing debate regarding the definition of csPCa. While this study chose ISUP grading group ≥ 2, different criteria for csPCa may yield different results. Lastly, this study is retrospective and conducted at a single center, which may introduce bias in the collection of information. Therefore, the conclusions drawn regarding PSAD cutoff values need further validation in prospective, multicenter clinical studies.

## Conclusion

Age, f/tPSA, and PSAD are independent predictors of diagnosing csPCa in patients with negative mpMRI. It is suggested that patients with negative mpMRI and PSAD less than 0.20 ng/ml/ml could avoid prostate biopsy, as a PSAD cutoff value of 0.20 ng/ml/ml has better diagnostic performance than the traditional cutoff value of 0.15 ng/ml/ml. This study provides data support for clinical decision-making regarding whether to perform prostate biopsy in Chinese patients with negative mpMRI.

### Electronic supplementary material

Below is the link to the electronic supplementary material.

Supplementary Material 1

## Data Availability

The data in this study are available from the author for correspondence upon reasonable request.
