# Supplementary material for: Risk factor analysis and optimal cutoff value selection of PSAD for diagnosing clinically significant prostate cancer in patients with negative mpMRI: results from a high-volume center in Southeast China
Source: World J Surg Oncol. 2024 May 28;22:140. doi: 10.1186/s12957-024-03420-7 (PMC11131245; doi:10.1186/s12957-024-03420-7)
Supplement: Supplementary file 1 — Supplementary Material 1 [file 12957_2024_3420_MOESM1_ESM.pdf]

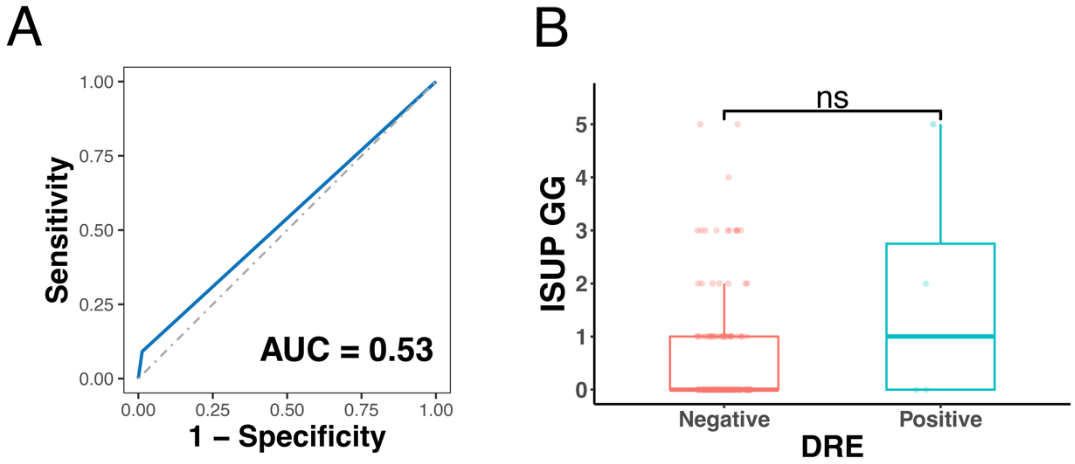

Supplementary Figure 1 DRE prediction performance in mpMRI-negative patients with csPCa.

A. ROC curve analysis for DRE prediction of csPCa. B. Boxplot of the association between DRE and ISUP GG.

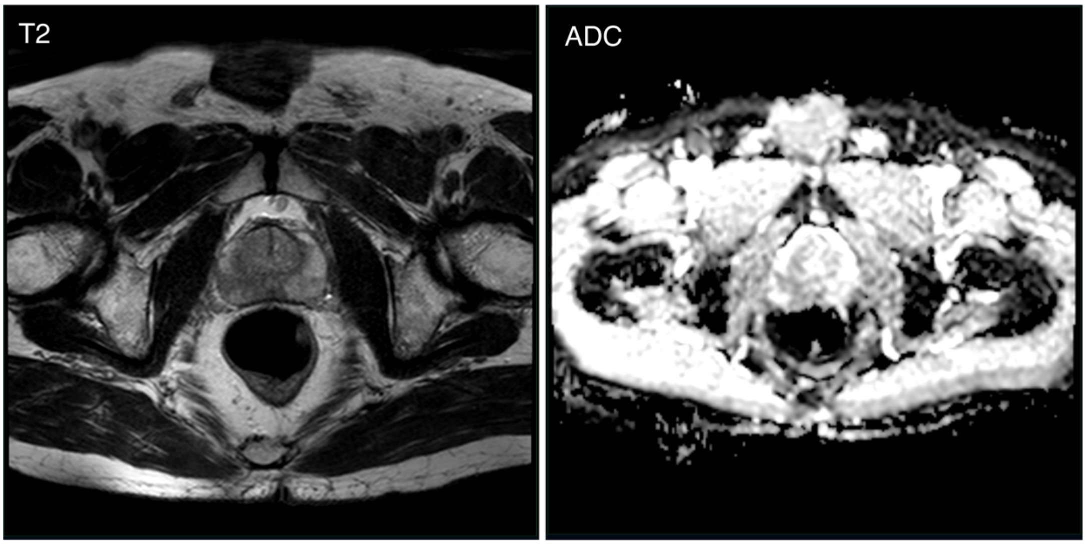

Supplementary Figure 2. Case: PI-RADS Score 2 with Gleason Score 4+3 (ISUP GG 3).

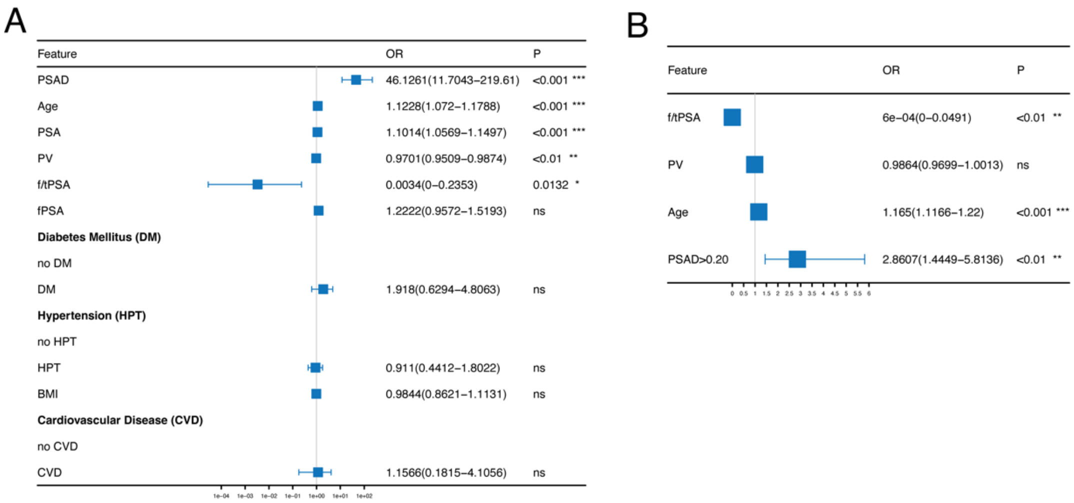

Supplementary Figure 3. Revised logistic regression analysis after excluding patients with PI-RADS > 2. A: Forest plot displaying the OR values of variables in univariate logistic regression. B. Forest plot displaying the OR values of variables in multivariate logistic regression.

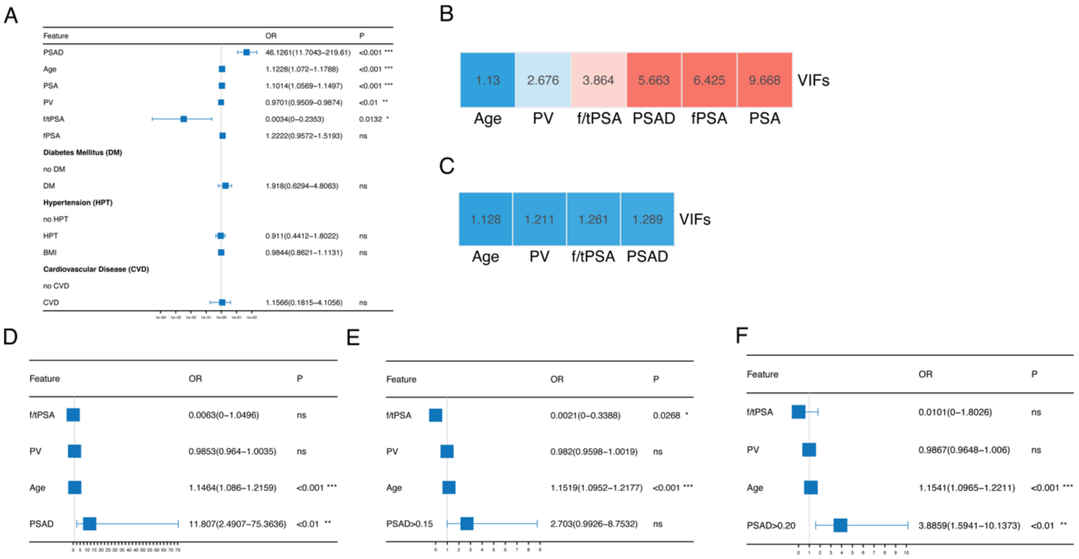

Supplementary Figure 4. Subgroup Analysis of Patients with ISUP GG > 2.

A. Univariate logistic regression analysis for predictors of ISUP GG > 2 csPCa in mpMRI negative patients. B. VIF analysis of significant variables in univariate analysis. C. VIF analysis after excluding collinear variables. D. Forest plot displaying the OR values of continuous variable PSAD in multivariate analysis. E. Forest plot displaying the OR values of PSAD > 0.15 ng/ml/ml in multivariate analysis. F. Forest plot displaying the OR values of PSAD > 0.20 ng/ml/ml in multivariate analysis.
